# High-pressure Raman scattering in bulk HfS_2_: comparison of density functional theory methods in layered MS_2_ compounds (M = Hf, Mo) under compression

**DOI:** 10.1038/s41598-018-31051-y

**Published:** 2018-08-24

**Authors:** J. Ibáñez, T. Woźniak, F. Dybala, R. Oliva, S. Hernández, R. Kudrawiec

**Affiliations:** 1 Institute of Earth Sciences Jaume Almera, Consell Superior d’Investigacions Científiques (CSIC), Lluís Solé i Sabarís s.n., 08028 Barcelona, Catalonia Spain; 2 Faculty of Fundamental Problems of Technology, Wroclaw University of Science and Technology, wybrzeże Wyspiańskiego 27, 50-370 Wrocław, Poland; 3 Departament d’Electrònica-MIND2-UB, Universitat de Barcelona, Martí i Franquès 1, 08028 Barcelona, Catalonia Spain

## Abstract

We report high-pressure Raman-scattering measurements on the transition-metal dichalcogenide (TMDC) compound HfS_2_. The aim of this work is twofold: (i) to investigate the high-pressure behavior of the zone-center optical phonon modes of HfS_2_ and experimentally determine the linear pressure coefficients and mode Grüneisen parameters of this material; (ii) to test the validity of different density functional theory (DFT) approaches in order to predict the lattice-dynamical properties of HfS_2_ under pressure. For this purpose, the experimental results are compared with the results of DFT calculations performed with different functionals, with and without Van der Waals (vdW) interaction. We find that DFT calculations within the generalized gradient approximation (GGA) properly describe the high-pressure lattice dynamics of HfS_2_ when vdW interactions are taken into account. In contrast, we show that DFT within the local density approximation (LDA), which is widely used to predict structural and vibrational properties at ambient conditions in 2D compounds, fails to reproduce the behavior of HfS_2_ under compression. Similar conclusions are reached in the case of MoS_2_. This suggests that large errors may be introduced if the compressibility and Grüneisen parameters of bulk TMDCs are calculated with bare DFT-LDA. Therefore, the validity of different approaches to calculate the structural and vibrational properties of bulk and few-layered vdW materials under compression should be carefully assessed.

## Introduction

Transition metal dichalcogenides (TMDCs) have attracted a great deal of attention during the last few years due to their remarkable properties and great potential for photonics and optoelectronics applications^1^. TMDCs are van der Waals layered semiconductors of the type MX_2_, where M stands for a transition metal atom such as Mo, W, or Hf, while X is a group-16 atom like S, Se, or Te. Due to their semiconducting character, TMDC monolayers emerge as an alternative to graphene, which does not normally exhibit an electronic bandgap. The optoelectronic properties (optical gaps and spin-orbit splittings) of TMDCs can indeed be tailored through strategically selecting their composition, i.e., alloying with different chalcogen or transition metal atoms^2^.

Despite a great deal of research has been devoted to the study of Mo and W-based TMDCs, relatively little attention has been paid to Hf-containing layered compounds. The latter are semiconductor materials with band gaps in the visible or infrared spectral range (the band gap decreases with increasing the chalcogen atomic number)^3^, which makes them attractive for a wide variety of optoelectronic devices like third-generation solar cells^4^. Recent advances on Hf-based TMDCs include the investigation of their promising thermoelectric properties^5–7^, their epitaxial growth on graphite or MoS_2_ substrates^8^, or the realization of different devices such as tunnel field-effect transistors (TFETs)^9^ or few-layer transistors^10^.

Among HfX_2_ compounds, hafnium disulphide (HfS_2_) has been found to exhibit particularly high and fast electrical responses^9,11^. It has been calculated that the mobility of his compound might reach values as high as 1800 cm^2^V^−1^s^−1^, much higher than the value of 400 cm^2^V^−1^s^−1^ of MoS_2_^11^. Also, it has been found that the sheet current density of tunneling field-effect transistors (TFETs) based on HfS_2_ is two orders of magnitude larger than that of MoS_2_^12^. It is thus necessary to further investigate the fundamental properties of HfS_2_ and explore its possible use in future device applications.

Raman spectroscopy is a powerful analytical tool which provides, in a non-destructive manner, valuable information about several important aspects of semiconductor materials and structures, such as their lattice dynamics, crystal quality, strain state, composition, or electronic structure. In the case of layered compounds such as graphene or TMDCs, Raman scattering is being widely used to determine flake thicknesses of few-layer systems and also to probe the strain, stoichiometry, or polytypism and stacking order of the TMDC layers^13,14^. However, in order to fully understand the vibrational properties of monolayer and few-layered TMDCs, it is essential to throroughly investigate the lattice dynamics of the buk materials.

High-pressure experiments are commonly employed in semiconductor physics studies in order to further probe the optical, electronic and vibrational properties of semiconductors. In particular, high-pressure techniques provide a highly useful benchmark to test the results of existing theoretical models, like for instance density functional theory (DFT), for the calculation of the electronic and lattice-dynamical properties of semiconductors. This may be critical in the case of TMDCs, whose properties are strongly affected by the weak van der Waals bonds between layers. In the case of Raman-scattering investigations, the pressure behavior of the optical phonons provides useful information about phase stability and anharmonic effects. In combination with theoretical calculations, it may also help one to assign the features that show up in the Raman spectra.

First-principle calculations have recently shown that, contrary to bulk HfS_2_, its monolayer form exhibits a phononic gap which can be tailored by applying biaxial strain^15^. So far, however, the high-pressure effects on the vibrational properties of bulk HfS_2_ have been investigated neither theoretically nor experimentally. With regard to ambient pressure conditions, several works dealing with the resonant and non-resonant Raman spectrum of bulk 1T-HfS_2_ have been published in the literature^16–18^. All the previous studies report the first-order *A*_1*g*_ and *E*_*g*_ Raman-active modes, but there is some discrepancy with regard to a weak feature that appears at ~325 cm^−1^ as a low-frequency shoulder of the intense *A*_1*g*_ peak. While some authors^16,18^ assign this feature to a Raman-forbidden *E*_*u*_(LO) mode, Roubi and co-workers^17^ attribute it to a forbidden A_2u_(LO) mode. It should be noted, however, that half the frequency of this mode (~162 cm^−1^) is very close to the frequency of the zone-center *E*_*u*_(TO) measured by Lucovsky and co-workers (166 cm^−1^) with infrared (IR) reflectance spectroscopy^19^. High-pressure measurements, in combination with lattice-dynamical calculations, could help to clarify the origin of all the Raman features and better assign these modes.

In the present work we report on Raman-scattering measurements under high hydrostatic pressures on bulk HfS_2_. From the pressure dependence of the Raman-active modes we obtain the corresponding mode Grüneisen parameters. The experimental pressure coefficients are compared with theoretical values obtained with lattice-dynamical ab initio calculations performed with density functional theory (DFT), using different functionals and methodologies. We find that the best agreement between theory and experiment is found with DFT-GGA calculations including Van der Waals interaction. In contrast, we show that bare DFT-LDA, which is widely used to calculate the properties of 2D (bulk and few-layer) compounds at ambient conditions, does not correctly predict the behavior of bulk HfS_2_ upon compression. Similar conclusions are reached for the case of 2H-MoS_2_.

## Results and Discussion

Three different types of calculations were performed with ABINIT in order to find the best agreement between theoretical and experimental lattice constants of HfS_2_ at ambient pressure: (i) calculations within the generalized gradient approximation (GGA), using fully-relativistic projector augmented-wave (PAW) potentials with Perdew-Burke-Ernzerhof (PBE) exchange-correlation functionals^20^; (ii) PAW-PBE calculation including Grimme’s D3 dispersion correction^21^ to take into account long-range van der Waals (vdW) interactions; (iii) calculations within the Perdew-Wang local density approximation (LDA); DFT-LDA usually provides successful structural results in layered compounds because of a compensating effect between the overestimated covalent part of the interlayer bonding and the neglected vdW forces^14^.

At ambient pressure, we obtained the following lattice constants for GGA calculations with (and without) vdW corrections: *a* = 3.616 (3.650) Å and *c* = 5.801 (7.021) Å. While the bare GGA-PBE calculations clearly overestimate the *c* parameter, the vdW-corrected GGA results are in very good agreement with the experimental values (*a* = 3.630 Å and 3.622, *c* = 5.854 and 5.88 Å)^19,22^, thus reflecting the importance of vdW interactions in the structural properties of these layered materials. On the other hand and, as expected, the LDA calculations provide somewhat underestimated values (*a* = 3.556 Å and *c* = 5.677 Å), although in reasonable agreement with the experimental data.

Table 1 shows a summary of these results, together with bulk modulus (*B*_0_) values obtained after structural relaxation at different pressures and subsequent fitting of the resulting volume-pressure data, using a 3^rd^ order Birch-Murnaghan equation of state. The Table also shows data for an additional calculation performed with the Quantum Espresso package using PBEsol functionals, i.e., the revised version of GGA-PBE for the solid state^23^. As can be seen in the table, a large compressibility (low bulk modulus) is obtained with the bare GGA calculations for HfS_2_, which is clearly a consequence of the overestimated *c* parameter at zero pressure. In turn, the PBEsol approach provides significantly improved results for the *c* parameter. However, a relatively large compressibility is still obtained with this functional. In contrast, similar bulk modulus values are found with GGA-vdW and LDA, although the latter yields a somewhat lower compressibility. This result may be attributed to the fact that the overestimation of the interlayer covalent bonding in LDA calculations becomes larger upon compression. Unfortunately, no X-ray diffraction measurements as a function of pressure have been so far reported for HfS_2_.

**Table 1:** Calculated lattice parameters at room pressure, bulk modulus and its pressure derivative for bulk HfS_2_ as obtained with different DFT functionals.

	*a* (Å)	*c* (Å)	*B*_0_ (GPa)	*B’*
LDA	3.556	5.677	36.8	8.3
PBE	3.650	7.021	8.1	8.3
PBE + vdW	3.616	5.801	30.6	8.3
PBEsol	3.588	5.943	19.4	12.7
Experiment	3.630^a^, 3.622^b^	5.854^a^, 5.88^b^	—	—

Experimental values for the room-pressure lattice parameters are also given.

^a^Ref.^19^, ^b^ref.^22^.

With regard to the vibrational properties, group theoretical analysis of the zone-center phonons of HfS_2_ with the ideal 1*T*-structure (*D*_3*d*_ point group, with 3 atoms in the primitive cell) shows that 9 vibrational modes are present in the total representation: *Γ* = *A*_1*g*_ + *E*_*g*_ + 2*A*_2*u*_ + 2*E*_*u*_, where the odd modes (*u*, ungerade) are infrared-active while the even modes (*g*, gerade) are Raman-active. Infrared and Raman activities are not compatible because the crystal structure has a center of inversion symmetry (and one of the modes for each of the *u*-symmetries correspond to acoustic modes). The atomic displacements for the optical modes can be found in previous works^15,17,19^.

The zero-pressure frequency values (*ω*_*i*0_), phonon pressure coefficients (*a*_*i*_) and mode Grüneisen parameters (*γ*_*i*_) obtained in this work with the Finite Displacement (FD) and Density Functional Perturbation Theory (DFPT) methods are shown in Tables 2 and 3. Table 2 shows the results for the gerade (g) modes of HfS_2_ (see below), while Table 3 shows the data for the TO and LO ungerade (u) phonons. The Grüneisen parameters were obtained using the expression *γ*_*i*_ = *B*_0_*a*_*i*_/*ω*_*i*0_, where, for the sake of comparison between different DFT methodologies, a constant value of *B*_0_ = 30.6 GPa as obtained from DFT calculations within GGA + vdW has been employed. These data will be later discussed in reference to the experimental results.

**Table 2:** Theoretical and experimental Raman frequencies (*ω*_*i*0_) and their pressure coefficients (*a*_*i*0_) and mode Grüneisen parameters (*γ*_*i*_) for the Raman-active (gerade) modes of HfS_2_.

Symmetry	Method	*ω*_*i*0_ (cm^−1^)	*a*_*i*0_ (cm^−1^GPa^−1^)	*γ*_*i*_
*A*_1 *g*_	FD (LDA)	332.9	4.88	0.45
	FD (PBE)	316.5	4.63	0.45
	FD (PBE + vdW)	330.7	4.63	0.43
	DFPT (LDA)	345.7	4.82	0.43
	DFPT (PBE)	321.3	4.42	0.42
	DFPT (PBEsol)	324.6	4.97	0.47
	Experiment^a^	340.2	4.71	0.42
*E*_*g*_	FD (LDA)	266.8	1.34	0.15
	FD (PBE)	243.7	2.87	0.36
	FD (PBE + vdW)	250.9	2.37	0.29
	DFPT (LDA)	270.2	1.80	0.20
	DFPT (PBE)	253.3	1.93	0.23
	DFPT (PBEsol)	259.1	1.79	0.21
	Experiment^a^	260.2	2.33	0.27

Theoretical values were obtained with the finite displacement (FD) method or within Density Functional Perturbation Theory (DFPT), using different functionals.

^a^This work.

**Table 3:** Theoretical and experimental Raman frequencies (*ω*_*i*0_) and their pressure coefficients (*a*_*i*0_) and mode Grüneisen parameters (*γ*_*i*_) for the Raman-inactive (ungerade) modes of HfS_2_.

Symmetry	Method	*ω*_*i*0_ (cm^−1^)	*a*_*i*0_ (cm^−1^GPa^−1^)	*γ*_*i*_
*A*_*2u*_(TO)	FD (LDA)	306.8	2.86	0.29
	FD (PBE)	302.8	2.27	0.23
	FD (PBE + vdW)	300.9	2.93	0.30
	DFPT (LDA)	324.3	2.82	0.27
	DFPT (PBE)	302.4	2.46	0.25
	DFPT (PBEsol)	306.9	2.71	0.27
	Experiment	—	—	—
*A*_*2u*_(LO)	FD (LDA)	324.4	3.05	0.29
	FD (PBE)	314.4	2.94	0.29
	FD (PBE + vdW)	317.8	3.25	0.31
	DFPT (LDA)	341.4	3.00	0.27
	DFPT (PBE)	318.5	2.80	0.27
	DFPT (PBEsol)	323.5	3.02	0.29
	Experiment^a^	321.1	3.58	0.34
*E*_*u*_(TO)	FD (LDA)	151.7	1.94	0.39
	FD (PBE)	137.8	3.15	0.70
	FD (PBE + vdW)	141.5	3.06	0.66
	DFPT (LDA)	173.8	1.75	0.31
	DFPT (PBE)	148.1	2.44	0.50
	DFPT (PBEsol)	152.2	2.23	0.45
	Experiment^b^	166	—	—
*E*_*u*_(LO)	DFPT (LDA)	308.4	0.30	0.03
	FD (LDA)	295.6	0.44	0.05
	FD (PBE)	290	1.5	0.16
	FD (PBE + vdW)	304.8	0.71	0.07
	DFPT (PBE)	301.1	0.57	0.06
	DFPT (PBEsol)	300.5	0.29	0.03
	Experiment^b^	318	—	—

Theoretical values were obtained with the finite displacement (FD) method or within Density Functional Perturbation Theory (DFPT), using different functionals.

^a^This work, ^b^ref.^19^.

**Figure 1:**
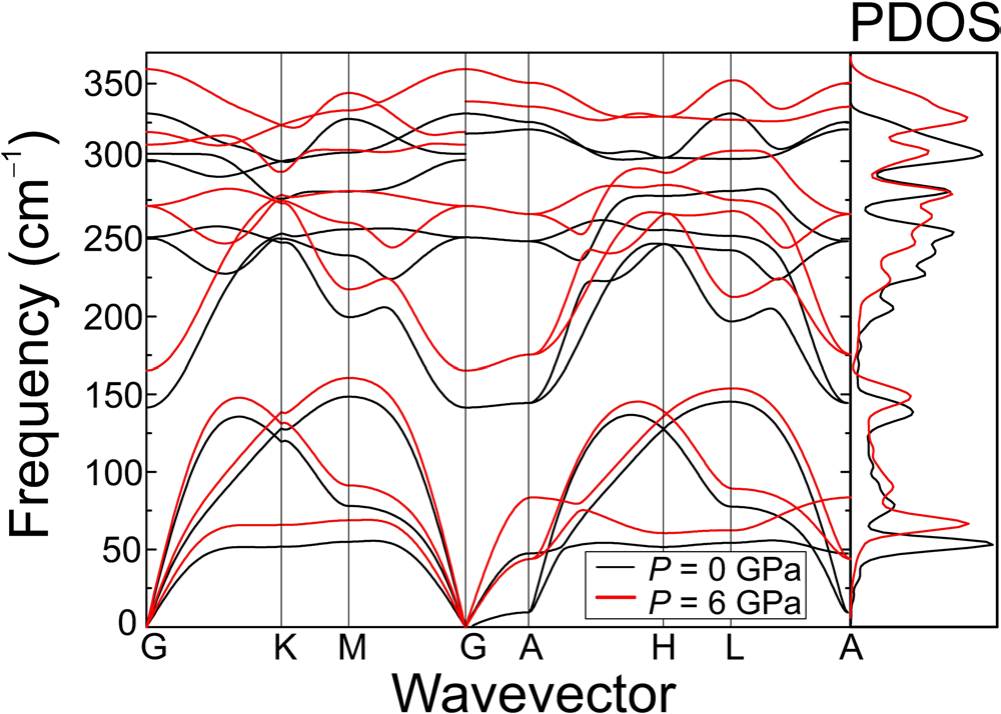
Ab initio calculation of the phonon band structure of bulk HfS_2_, including TO-LO splitting effects, along the main lines of symmetry at 0 and 6 GPa. The right panel shows the corresponding phonon density of states (PDOS).

Figure 1 shows the calculated phonon dispersion and one-phonon density of states (1-PDOS) over the whole Brillouin zone as obtained with Phonopy for HfS_2_ at 0 and 6 GPa, using the finite displacement (FD) method and a PBE functional including the vdW correction. The figure includes splitting between LO and TO modes, which was not taken into account in previous works^15^. It should be recalled, however, that DFT calculations tend to yield large errors in the case of LO frequencies as a consequence of the band-gap problem and the computation of the dielectric constants. This is particularly important in the case of DFT-LDA, although it is not substantially improved within GGA^24^.

The corresponding 1-PDOS curves are displayed in the right panel of the figure. As expected, all phonon branches show an overall upward frequency shift upon compression, without any softening of the modes. The results at zero pressure are consistent with those obtained by DFPT within PBE-GGA as reported in ref.^5^. As can be seen in the figure, no phononic gap exists in bulk HfS_2_, which suggests that reduced optical phonon lifetimes may be expected in this compound for all the first-order modes due to an increased number of pathways for phonon decay. Interestingly, many of the phonon branches, like for instance the acoustic phonons along Γ-K-M-Γ or the optical phonons along the Γ-A direction, exhibit very low dispersion and therefore increased two-phonon density of states. As a consequence of this, second-order Raman scattering processes may be favored in HfS_2_.

**Figure 2:**
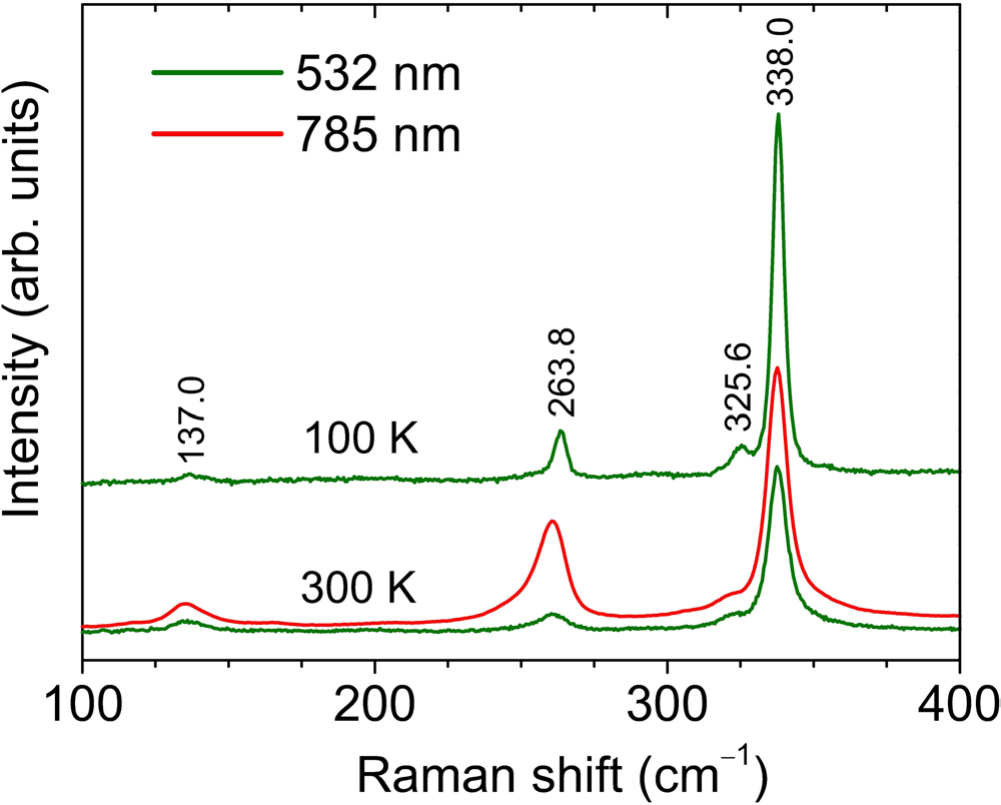
Room-pressure Raman spectra, acquired at room temperature and low temperature (100 K), excited with λ = 532 nm. For comparison, a Raman spectrum excited at room conditions with λ = 785 nm is also shown.

In order to evaluate the validity of different functionals and methodologies based on DFT calculations, Raman measurements were performed at different pressures and temperatures. Figure 2 shows ambient-pressure Raman spectra at room temperature and low temperature (100 K) of HfS_2_, acquired with 532-nm excitation radiation. An additional room-temperature spectrum excited with λ = 785 nm has also been included in the figure for comparison purposes. Four different modes around 137, 264, 326 and 338 cm^−1^ clearly show up in the low-temperature spectrum. The Raman peaks at ~338 and 264 cm^−1^ can be unambiguously assigned to the *A*_1*g*_ and *E*_*g*_ modes, respectively^16–18^. It is interesting to note that these modes only involve S vibrations, and as a consequence they are not affected by isotopic anharmonic effects due to the strong isotopic variability of natural hafnium. In contrast, strong isotopic broadening may be expected for the *u*-phonons, since these do involve Hf vibrations.

As expected, all the features in Fig. 2 are broadened and shifted to lower frequencies in the room-temperature spectra. In particular, the mode at ~325 cm^−1^ only appears as a broad low-frequency shoulder of the *A*_1*g*_ mode. In turn, it is worth noting that the *E*_*g*_ mode at ~264 cm^−1^ is significantly enhanced, relatively to the rest of peaks, in the spectrum excited with λ = 785 nm. As in other layered compounds^25–27^, this intensity enhancement may be attributed to resonance effects arising from exciton-phonon coupling involving particular exciton states of HfS_2_. Given that atoms in the *E*_*g*_ phonon vibrate in-plane, the observed intensity increase in Fig. 2 may be ascribed to electronic transitions involving excitonic states with strong in-plane character. This is what occurs, for instance, is the case of the C exciton in MoS_2_^25^. However, given that the combination modes in TMDCs exhibit strong resonance enhancement due to coupling between phonons of nonzero momentum and excitonic transitions^27,28^, it cannot be completely ruled out that the strong peak that appears at ~264 cm^−1^ for 785-nm excitation has contribution from a second-order Raman band. More work is required to fully understand excitonic resonance Raman effects in HfS_2_.

In spite of the Raman inactivity of *u*-modes, two different works^16,18^ attribute the weak peak below the *A*_1*g*_ mode around 326 cm^−1^ to an *E*_*u*_(LO) mode. In contrast, ref.^17^ assigns this feature to an *A*_2*u*_(LO) mode. In that work, a weak, broad band around 155 cm^−1^ is also attributed to an *E*_*u*_(TO) mode. However, such feature in not reported in any other previous work, whereas the *E*_*u*_(TO) phonon was found at 166 cm^−1^ by means of infrared-reflectance spectroscopy^19^. Note that the peak at 326 cm^−1^ could also correspond to a 2*E*_*u*_(TO) mode, since this feature is not far from twice the frequency of the *E*_*u*_(TO) mode reported by Lucovsky and co-workers.

Leaving aside the previous works on HfS_2_, several studies on layered compounds like MoS_2_, MoSe_2_ or WS_2_ have also reported Raman inactive *u*-modes^27–29^. In those works, ungerade modes were observed amid a large number of second-order modes excited resonantly, and were attributed to symmetry breaking of the selection rules through disorder or the participation strongly localized electronic states. Other works on TMDCs reported bulk-inactive *g*-modes in few-layer compounds like MoTe_2_^30^, which was attributed to a loss of periodicitiy along the *c*-axis. In the case of HfS_2_, it cannot be ruled out that the *u*-modes show up in the Raman spectra due to a contribution of few-layer domains in the investigated samples. Regardless of their origin, high-pressure Raman-scattering measurements, in combination with DFT lattice-dynamical calculations, may provide highly valuable information to identify the different features that appear in the Raman spectra of vdW compounds.

**Figure 3:**
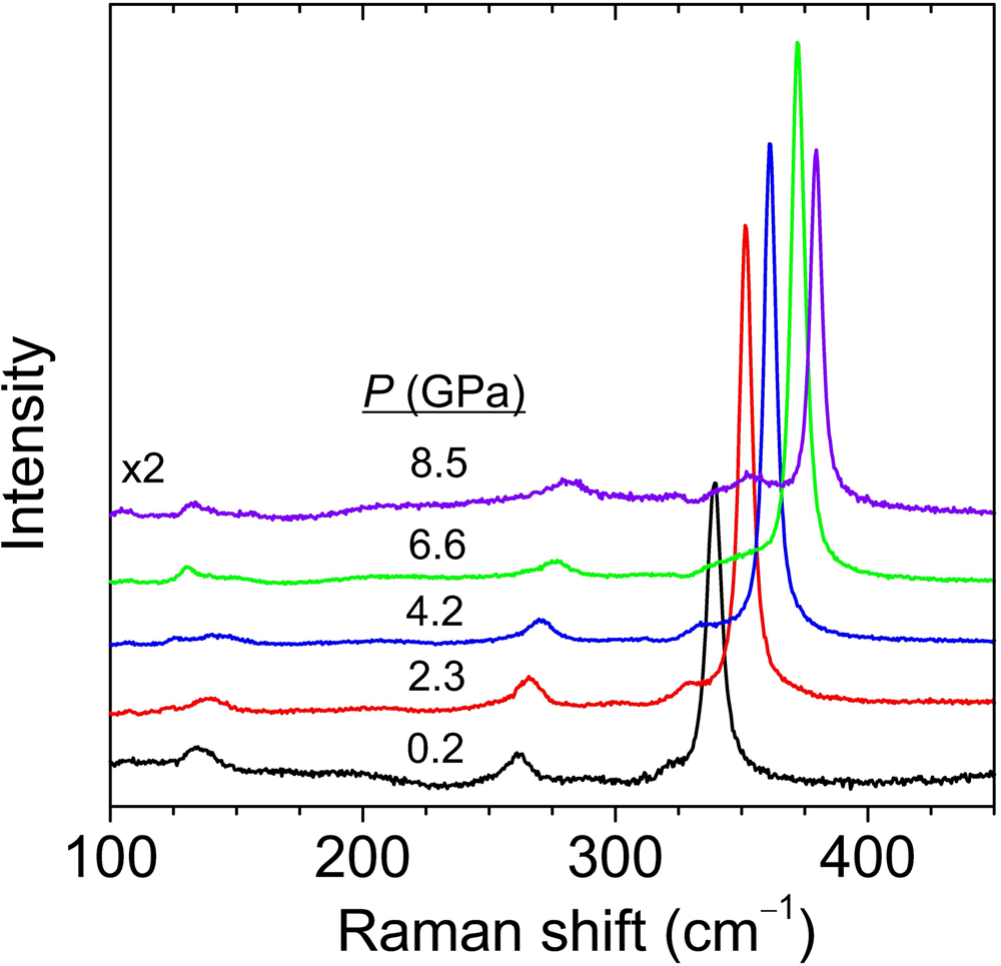
Raman spectra of bulk HfS_2_ acquired at different hydrostatic pressure values up to 8.5 GPa.

**Figure 4:**
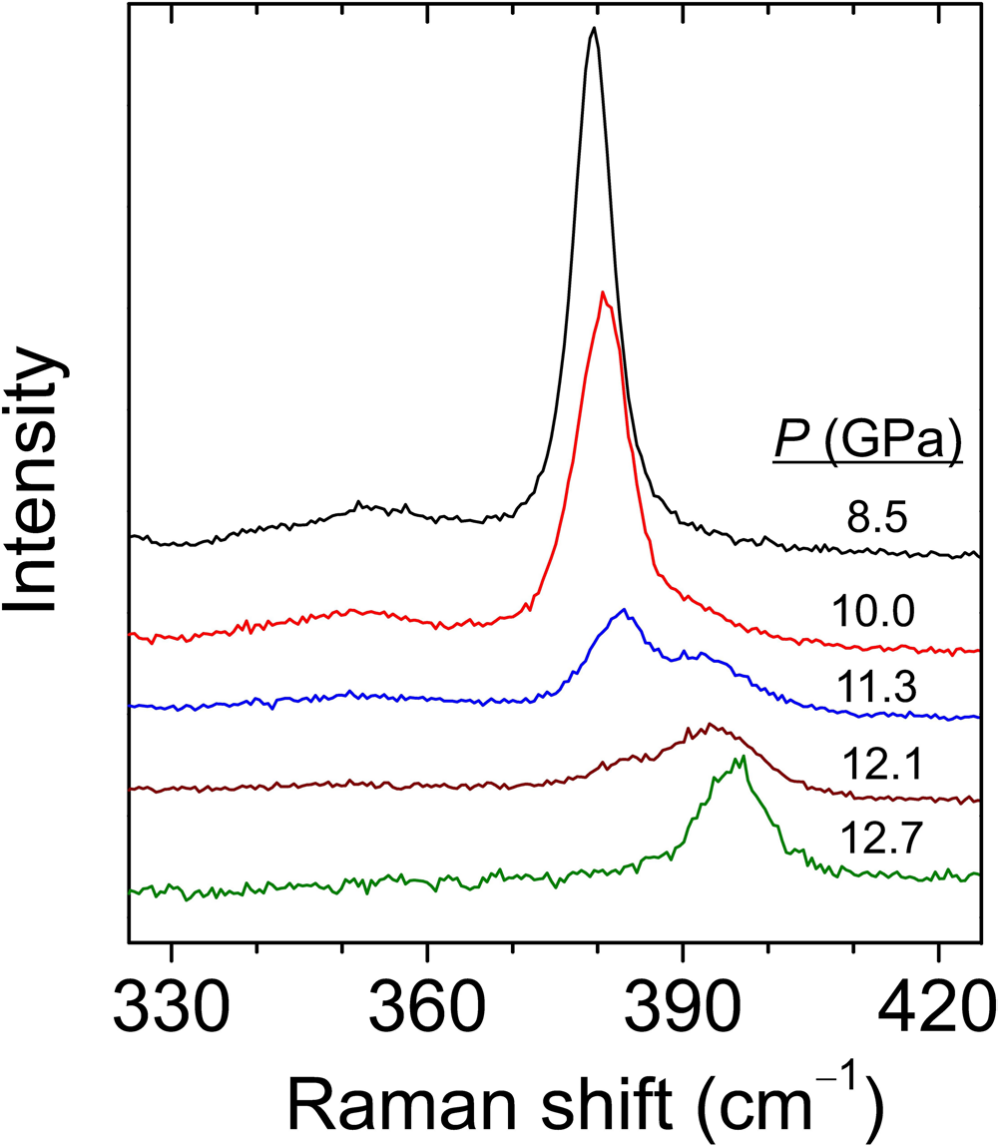
Raman spectra of bulk HfS_2_ acquired at different hydrostatic pressure values, up to 12.7 GPa, showing the onset of a possible first-order phase transition in this compound at around 11 GPa.

Figure 3 shows selected Raman spectra at different pressures, up to 8.5 GPa, for bulk HfS_2_. As expected, all modes are found to blueshift with increasing pressure. Around 4 GPa, a very weak, broad feature is found to show up around 120 cm^−1^. This mode has never been reported in previous works and probably emerges due to a particular resonant excitation at this pressure range (note that, as can be seen in Fig. 1, 1T-HfS_2_ has no first-order optical modes below ~150 cm^−1^). As already mentioned, second-order resonant Raman scattering in TMDCs may yield a large number of bands at some given excitation conditions. In this case, resonance enhancement may be achieved by pressure-induced bandgap shifts. In contrast, the mode at 137 cm^−1^ progressively weakens upon compression and is hardly visible above 6 GPa. Around 10 GPa, as shown in Fig. 4, a new peak appears above the *A*_1*g*_ mode. This peak becomes dominant at 12.7 GPa and can be tentatively attributed to a high-pressure phase of HfS_2_. The study of this possible new phase is beyond the scope of the present work.

**Figure 5:**
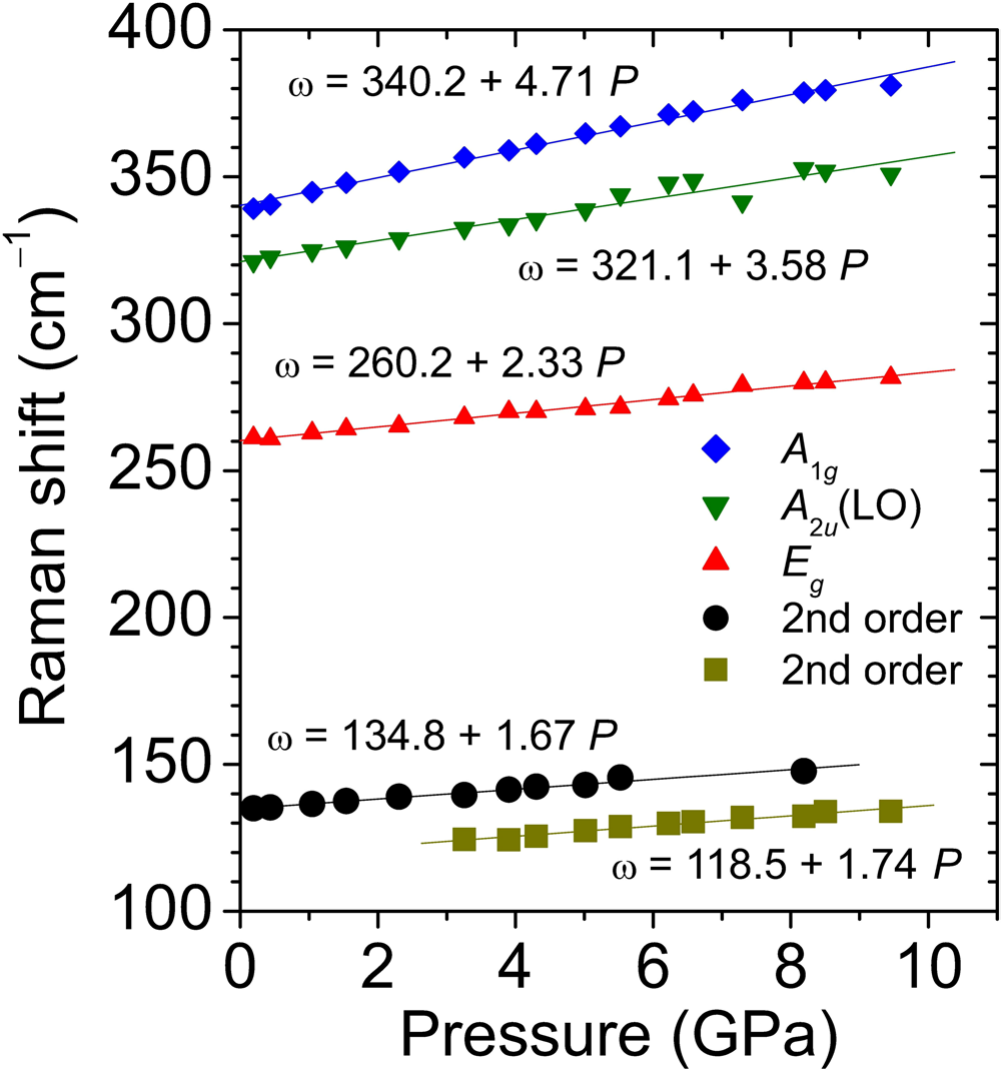
Pressure dependence of first-order optical phonons and second-order bands that appear in the Raman spectrum of bulk HfS_2_. The lines show the result of linear fits to the experimental data.

We have plotted in Fig. 5 the pressure dependence of all the observed Raman features up to 10 GPa, below the phase transition that presumably occurs in bulk HfS_2_. The results of linear fits to all the experimental curves as a function of pressure (*P*) are also shown in the figure. These where obtained by using the expression *ω*_*i*_(*P*) = *ω*_*i*_(0) + *a*_*i*_*P*, where *ω*_*i*_(0) and *a*_*i*_ stand for the zero-pressure frequency and pressure-coefficient for the *i*-th mode, respectively. The experimental data are summarized in Tables 2 and 3, together with theoretical values obtained within DFT-based lattice-dynamical calculations of the zone-center phonons performed with the FD and DFPT methods. The corresponding mode Grüneisen parameters (*γ*_*i*_ = *B*_0_*a*_*i*_ /*ω*_*i*0_, with *B*_0_ = 30.6 GPa as obtained with the present DFT-GGA calculations including vdW interactions) are also given.

In the case of the *A*_1*g*_ mode, excellent agreement is found between the experimental pressure coefficients and most of the approaches considered in this work. For this phonon mode, with atoms vibrating out-of-plane, all methods provide similar results because interlayer forces have a low effect on the resulting frequencies of vibration. As expected, among the DFPT values (note that vdW corrections are not yet implemented for DFPT phonon calculations in Quantum Espresso), LDA provides the best zero-pressure frequency value. In the case of FD calculations, LDA and GGA + vdW provide comparable results. The bare GGA value, however, yields a too low value as a consequence of the overestimated *c* parameter at zero pressure.

Similar conclusions can be reached for the IR-active *A*_2*u*_(TO) mode, although in this case the bare GGA calculations tend to yield slightly reduced pressure coefficients. In turn, DFPT-LDA predicts a zero-pressure frequency that is ~6–7% larger than the rest of values. Unfortunately, no experimental results are available to perform a comparison between theory and experiment for this mode.

Interestingly, when the zone-center modes involve in-plane atomic displacements, as is the case of *E*_*g*_ and *E*_*u*_ modes, the LDA calculations predict fairly low pressure coefficients, below 2 cm^−1^/GPa, both with the FD and DFPT methods. In contrast, the GGA and GGA-vdW results predict much larger values. In particular, the GGA-vdW pressure coefficient obtained with the FD method for the *E*_*g*_ mode (2.37 cm^−1^/GPa) is in remarkable agreement with the experimental value (2.33 cm^−1^/GPa). This result indicates that the GGA-vdW calculations are better suited to model the high-pressure lattice-dynamical properties of 1T-HfS_2_.

Such a conclusion is not limited to the case of HfS_2_, but can be extended to other layered compounds. To further test this point, we have performed additional lattice-dynamical calculations for the case of the archetypical TMDC compound 2H-MoS_2_ (see the results in Supplementary Table 1). In this case we also find that, although the LDA functional predicts remarkable zero-pressure Raman frequencies, the theoretical pressure coefficients for the *E*-symmetry modes, including the low-frequency shear mode, are significantly lower than those obtained with PBE functionals (with and without vdW corrections). Bearing in mind the relatively large dispersion of the experimental data reported in the literature (Supplementary Table 1), only the GGA-vdW calculations provide good agreement between theoretical and experimental values for both the zero-pressure Raman frequencies and their corresponding phonon pressure coefficients in bulk MoS_2_. Similar conclusions were also reached elsewhere^31^ for the case of black phosphorous. In that work, although no direct comparison was shown regarding different methods to calculate the lattice dynamics of this layered material as a function of pressure, it was found that only the PBE-vdW calculations are able to simultaneously predict the structural and vibrational properties of black phosphorous under compression.

On the other hand, and as shown in Table 3, the theoretical pressure coefficients for the *E*_*u*_(LO) mode of bulk HfS_2_ are found to be very small, much lower than 1 cm^−1^/GPa. This result, together with the theoretical *ω*_*i*0_ values found for this mode, allow us to discard the conclusions of refs^16,18^ with regard to the origin of the peak at ~320 cm^−1^ (Fig. 2). In those works, this feature was attributed to an *E*_u_(LO) mode. In contrast, the experimental pressure coefficient (zero-pressure frequency) for this band is 3.58 cm^−1^/GPa (321.1 cm^−1^), much larger than the values predicted by the different calculations for the *E*_*u*_(LO) mode, e. g. 0.71 cm^−1^/GPa (304.8 cm^−1^) with GGA + vdW and the FD method. Note also that the *E*_u_(LO) mode should involve in-plane vibrations and, following the behavior of the *E*_*g*_ mode, resonant enhancement of this mode would have been expected for 785-nm excitation. In contrast, the theoretical values for the out-of-plane *A*_2*u*_(LO) [3.25 cm^−1^/GPa (317.8 cm^−1^) with GGA + vdW and the FD method] are compatible with the experimental values. Note also that no combinations of first-order modes at different points of the Brillouin zone, using the phonon dispersion of Fig. 1, provide a satisfactory enough explanation for this feature. In particular, it cannot be attributed to a 2*E*_*u*_(TO) mode, since all the present DFT calculations predict a much larger pressure coefficient for any second-order combination of optical modes. Therefore, given the good agreement between the experimental and theoretical (GGA + vdW) values, we conclude that the low-frequency shoulder of the *A*_1*g*_ mode can be assigned to the silent *A*_2*u*_(LO) phonon of HfS_2_. More work would be necessary, however, to fully understand the actual mechanism giving rise to silent (symmetry-forbidden) modes in HfS_2_ and, in general, in layered TMDCs.

Finally, we use the theoretical phonon dispersion as a function of pressure (Fig. 1) in combination with the experimental pressure coefficients in order to tentatively assign the remaining Raman feature at 137 cm^−1^ as well as the weak band that shows up above 3 GPa (Fig. 2). First, we note that the experimental pressure coefficient of the latter, and also that of the feature at 137 cm^−1^ is just around 1.7 cm^−1^/GPa, much smaller than the value predicted for the *E*_*u*_(TO) mode (Table 3). Hence, in contrast to the conclusions of ref.^17^, it can be ruled out that forbidden first-order Raman scattering by the *E*_*u*_ (TO) mode occurs in HfS_2_. Indeed, given that numerous phonon branches display low dispersion along relative large regions of the Brillouin zone (see Fig. 1), and bearing in mind the low experimental pressure coefficients of the observed bands, it is very likely that these two modes correspond to second-order difference modes. For instance, combinations like *Eg*(L)-LA(L) or *A*_2*u*_(TO,L)-*E*_*u*_(TO,L) might explain the pressure behavior of the peak at 137 cm^−1^, while the band that shows up above 3 GPa at 118 cm^−1^ can be explained with a *E*_*u*_(TO,M)-LA(M) or a *E*_*u*_(TO,L)-LA(L) combination.

## Conclusion

We have presented a joint theoretical and experimental high-pressure Raman-scattering study of the vibrational properties of bulk 1T-HfS_2_. Comparison between the experimental phonon pressure coefficients and the results of DFT calculations with different functionals, with and without vdW interactions, indicate that GGA + vdW properly describes the pressure behavior of bulk HfS_2_. In contrast, it is found that bare DFT-LDA fails to reproduce the structural and vibrational properties of this compound under compression. Similar conclusions can be reached in the case of 2H-MoS_2_. DFT-LDA is widely used for calculations of phonons and other properties in layered compounds due to a well-known compensating effect between the underestimated bond lengths and the overestimated interatomic forces. However, the present results suggest that DFT-LDA is not valid to predict the compressibility or the mode Grüneisen parameters of bulk or few-layered vdW materials. In particular, we have found that DFT-LDA gives rise to a sizable underestimation of phonons involving in-plane atomic displacements. From the pressure dependence of several Raman features and the theoretical data, it has been possible to shed additional light on the origin of different features that appear in the Raman spectrum of HfS_2_. In particular, the low-frequency shoulder below *A*_1*g*_ peak has been assigned to a forbidden *A*_2*u*_(LO) mode. More work is required in order to understand the scattering mechanism responsible for the appearance of Raman-inactive ungerade modes in the Raman spectrum of TMDCs.

## Methods

### Samples

For the present study, we used a commercial HfS_2_ bulk sample from 2DSemiconductors Inc. grown by the Bridgman technique. The indirect and direct optical band-gap of the sample, as measured by optical measurements at ambient conditions, are 1.39 and 2.09 eV, respectively^3^.

### High pressure measurements

Small flakes of around 50 × 50 μm^2^ were detached from the bulk HfS_2_ sample and loaded into a gasketed membrane-type diamond anvil cell (DAC) with 400 µm culet-size diamonds. A mixture of methanol-ethanol-water (16:3:1) was employed as pressure transmitting medium, and the ruby fluorescence method was used to evaluate the pressure applied to the flake. Room-temperature micro-Raman measurements were acquired during the upstroke cycle, up to 13 GPa, by using a HORIBA Jobin-Yvon LabRam-HR spectrometer coupled to a high-sensitive CCD camera. The spectra were excited with the second harmonic of a continuous-wave Nd:YAG laser (λ = 532 nm), and a ×50 long-working distance objective was employed to focus the laser light and to collect the backscattered radiation. To further probe resonance effects in HfS_2_ at room temperature, additional Raman spectra excited with λ = 785 nm radiation were also acquired.

### First-principle calculations

DFT calculations for bulk 1T-HfS_2_ and 2H-MoS_2_ were carried out with the ABINIT^32^ and Quantum Espresso^33^ packages. Details for the case of MoS_2_ can be found in the Supplementary Material. For all calculations on HfS_2_, the plane wave basis cut-off was 30 Ha and the Monkhorst-Pack *k*-point grid was set to 8 × 8 × 4. The structures were first fully relaxed until the interatomic forces were lower than 10^−5^ eV/Å. Lattice-dynamical calculations were performed with the Phonopy software^34^ for different pressure values, up to 10 GPa, within the FD method. 2 × 2 × 2 supercells were found to give convergence for all the phonon modes of both compounds. From these calculations, zone-center phonon frequencies for the Raman-active *A*_1*g*_ and *E*_1*g*_ modes and for the infrared-active *A*_2*u*_ and *E*_*u*_ modes of HfS_2_ were obtained. For the construction of the corresponding dynamical matrices, the forces associated to the selected finite displacements were calculated with ABINIT using the GGA, GGA-vdW and LDA methods. In order to calculate the splitting between transverse optical (TO) and longitudinal optical (LO) phonon modes, dielectric tensors and Born effective charges were calculated as a function of pressure with Quantum Espresso, using the DFPT approach implemented in this package. For this purpose, LDA and PBEsol functionals were used. For comparison purposes, zero-pressure frequencies and pressure coefficients for the zone-center modes including TO-LO splitting were also calculated within DFPT.

## Electronic supplementary material

Supplementary material
