# Supplementary material for: High-pressure Raman scattering in bulk HfS2: comparison of density functional theory methods in layered MS2 compounds (M = Hf, Mo) under compression
Source: Sci Rep. 2018 Aug 24;8:12757. doi: 10.1038/s41598-018-31051-y (PMC6109144; doi:10.1038/s41598-018-31051-y)
Supplement: Supplementary file 1 — Supplementary material [file 41598_2018_31051_MOESM1_ESM.docx]

**Supplementary material: High-pressure Raman scattering in bulk HfS_2_: comparison of density functional theory methods in layered MS_2_ compounds (M = Hf, Mo) under compression**

J. Ibáñez^1^, T. Woźniak^2^, F. Dybala^2^, R. Oliva^2^, S. Hernández^3^, R. Kudrawiec^2^

^1^Institute of Earth Sciences Jaume Almera, Consell Superior d’Investigacions Científiques (CSIC), Lluís Solé i Sabarís s.n., 08028 Barcelona, Catalonia, Spain

^2^Faculty of Fundamental Problems of Technology, Wroclaw University of Science and Technology, wybrzeże Wyspiańskiego 27, 50-370 Wrocław, Poland

^3^Departament d’Electrònica-MIND2-UB, Universitat de Barcelona, Martí i Franquès 1, 08028 Barcelona, Catalonia, Spain

Numerous works have reported experimental pressure coefficients for the optical phonons of bulk 2H-MoS_2_, obtained with high-pressure Raman measurements. Taking advantage of this, and to test the hypothesis that DFT-LDA calculations tend to underestimate the pressure coefficients of the in-plane modes of TMDCs, we have performed additional DFT lattice-dynamical calculations using different methods: i) finite displacement (FD) with Phonopy (see Methods section), relaxing the lattice with ABINIT and using LDA and PBE functionals (with and without van der Waals D3 corrections); ii) density functional perturbation theory (DFPT) calculations with Quantum Espresso, using LDA and PBE functionals. For these calculations, a cutoff energy of 20 Ha and a 12x12x4 *k*-point grid were used. The convergence criteria were the same as those employed for HfS_2_.

As is well known, group theoretical analysis of the zone-center phonons of 2H-MoS_2_, with 6 atoms in the primitive cell, yields the following total representation: *Γ* = *A*_1_*_g_* + 2*A*_2_*_u_* + 2*B*_2_*_g_* + *B*_1_*_u_* + *E*_1_*_g_* + 2*E_1u_* + 2*E_2g_* + *E*_2_*_u_*, where only the gerade modes (*A*_1_*_g_*, *E*_1_*_g_* and *E*_2_*_g_*) are Raman-active optical modes. *E*-symmetry modes are doubly degenerate, and one of the two *E*_2_*_g_*, namely E^2^_2_*_g_*, corresponds to the low-frequency shear mode; the optical modes can be grouped in Davydov pairs [see for instance Gołasa *et al.*, Solid State Commun. **197**, 53 (2014)] which are nearly degenerate (the small frequency splitting of such modes is a consequence of weak interlayer forces).

Table I shows the results of different DFT lattice-dynamical calculations performed in this work for the Raman-active modes of bulk 2H-MoS_2_. All calculations showed a quadratic dependence, also observed in previous experimental works [see for instance Sugai and Ueda, Physical Review B **26**, 6554 (1982)] and therefore the pressure coefficients were obtained with a quadratic fit to the theoretical data. Experimental data from three different Raman studies is also given for comparison purposes. As can be seen in the table, the LDA pressure coefficients for the doubly-degenerate *E*-symmetry modes of 2H-MoS_2_, involving in-plane vibrations, tend to be sizably lower than those obtained with PBE functionals. PBE+vdW, however, also seems to underestimate the experimental frequency of the *A*_1_*_g_* mode, the frequency of which is well reproduced by the bare PBE calculations. Overall, only the results obtained with PBE including vdW corrections are in sufficiently good agreement with the experimental results both at zero pressure and under compression. While LDA clearly underestimates the pressure coefficients and bare PBE fails at ambient pressure, PBE+vdW provides the most reasonable prediction of the structural and vibrational properties of 2H-MoS_2_ at zero pressure and under compression.

| Symmetry | Method | *ω_i_*_0_ (cm^−1^) | *a_i_*_0_ (cm^−1^GPa^−1^) |
| --- | --- | --- | --- |
|  | FD (LDA) | 35.3 | 3.45 |
|  | FD (PBE) | 25.1 | 4.23 |
|  | FD (PBE+vdW) | 29.7 | 3.73 |
| *E*^2^_2_*_g_* | DFPT (LDA) | 35.3 | 3.57 |
|  | DFPT (PBE) | 20.8 | 4.58 |
|  | Experiment^a^ | 33.5 | 4.5 |
|  | Experiment^b^ | − | − |
|  | Experiment^c^ | 32 | − |
|  | FD (LDA) | 290.1 | 1.80 |
|  | FD (PBE) | 277.7 | 2.02 |
|  | FD (PBE+vdW) | 282.2 | 1.94 |
| *E_1g_* | DFPT (LDA) | 289.3 | 1.47 |
|  | DFPT (PBE) | 279.2 | 1.63 |
|  | Experiment^a^ | 287 | − |
|  | Experiment^b^ | − | − |
|  | Experiment^c^ | 287 | − |
|  | FD (LDA) | 388.1 | 1.37 |
|  | FD (PBE) | 374.3 | 1.80 |
|  | FD (PBE+vdW) | 378.2 | 1.76 |
| *E*^1^_2_*_g_* | DFPT (LDA) | 388.1 | 1.32 |
|  | DFPT (PBE) | 375.6 | 1.97 |
|  | Experiment^a^ | 387 | 1.9 |
|  | Experiment^b^ | 383.1 | 1.80 |
|  | Experiment^c^ | 383 | 1.73 |
|  | FD (LDA) | 413.6 | 3.26 |
|  | FD (PBE) | 399.5 | 3.41 |
|  | FD (PBE+vdW) | 406.5 | 3.08 |
| *A*_1_*_g_* | DFPT (LDA) | 412.7 | 3.21 |
|  | DFPT (PBE) | 401.6 | 3.99 |
|  | Experiment^a^ | 413 | 4.0 |
|  | Experiment^b^ | 408.2 | 3.60 |
|  | Experiment^c^ | 409 | 3.90 |

^a^Sugai and Ueda, Physical Review B **26**, 6554 (1982)

^b^Livneh and Sterer, Phys. Rev. B **81**, 195209 (2010)

^c^Bhatt *et al.*, J. Raman Spectrosc. **45**, 971 (2014)

**Table I.** Theoretical and experimental Raman frequencies (*ω_i_*_0_) and their pressure coefficients (*a_i_*_0_) for the Raman-active modes of bulk 2H-MoS_2_. Theoretical values were obtained with the finite displacement (FD) method or within Density Functional Perturbation Theory (DFPT), using different functionals.
